# Interaction of Streaming and Attention in Human Auditory Cortex

**DOI:** 10.1371/journal.pone.0118962

**Published:** 2015-03-18

**Authors:** Alexander Gutschalk, André Rupp, Andrew R. Dykstra

**Affiliations:** Department of Neurology, Ruprecht-Karls-Universität Heidelberg, Heidelberg, Germany; Kyoto University, JAPAN

## Abstract

Serially presented tones are sometimes segregated into two perceptually distinct streams. An ongoing debate is whether this basic streaming phenomenon reflects automatic processes or requires attention focused to the stimuli. Here, we examined the influence of focused attention on streaming-related activity in human auditory cortex using magnetoencephalography (MEG). Listeners were presented with a dichotic paradigm in which left-ear stimuli consisted of canonical streaming stimuli (*ABA_* or *ABAA*) and right-ear stimuli consisted of a classical oddball paradigm. In phase one, listeners were instructed to attend the right-ear oddball sequence and detect rare deviants. In phase two, they were instructed to attend the left ear streaming stimulus and report whether they heard one or two streams. The frequency difference (ΔF) of the sequences was set such that the smallest and largest ΔF conditions generally induced one- and two-stream percepts, respectively. Two intermediate ΔF conditions were chosen to elicit bistable percepts (i.e., either one or two streams). Attention enhanced the peak-to-peak amplitude of the P1-N1 complex, but only for ambiguous ΔF conditions, consistent with the notion that automatic mechanisms for streaming tightly interact with attention and that the latter is of particular importance for ambiguous sound sequences.

## Introduction

A major challenge for our central auditory system is to segregate simultaneous streams of auditory information that we receive from two or more sound sources. Sequence parameters which determine whether sequentially presented tones are grouped into the same stream have been explored with the so-called *streaming* paradigm (aka *stream segregation*) [1]. This paradigm typically uses sequential tone patterns of two or more different tones (e.g., *A* and *B*). A number of physical parameters influence whether a sequence is perceived as one integrated stream of alternating tones or as two segregated monotone sequences (e.g. inter-tone interval [2], frequency separation, or ΔF [3,4], and several others [5]).

An ongoing debate in the field is the extent to which focused attention influences the streaming process. Bregman [1] suggested that primitive streaming cues—such as ΔF—could promote streaming at an early processing stage without requiring attention or other top-down mechanisms. This hypothesis is supported by EEG studies which found that the occurrence of the mismatch negativity (MMN), a component of the auditory evoked response elicited by a change in an otherwise regular stimulus sequence [6], depends on the organization of auditory streams [7,8]. Moreover, the transient waves P_1_m and N_1_m evoked by each tone of a sequence increase in amplitude with frequency separation [9,10], probably as a consequence of selective adaptation [11]. This ΔF dependent modulation of the P_1_m and N_1_m covaries with listeners’ rating of streaming perception. All of these evoked response components—the P_1_m, N_1_m, and MMN—can be recorded while listeners are not engaged in the auditory stimulation and even when attention is focused on another task.

However, Carlyon et al. [12] challenged Bregman’s view and argued that streaming requires focused attention towards the source to be segregated. These authors used dichotic stimuli and instructed listeners to first listen to their right ear and perform an unrelated distractor task while an *ABA_* streaming sequence played in the left ear. Listeners were further instructed to switch their attention to the left ear after the cessation of the distracting task and report their streaming percept of the *ABA_* triplets. The results showed that streaming was not stable at the time of the attention switch, but rather required a new “build-up” period [13,14]. When listeners rated streaming from the beginning of the sequence, in contrast, streaming was at a constantly high level after identical time delays. Moreover, patients with neglect after right-hemisphere brain lesions, who have an attentional deficit for left-sided stimuli, showed lower streaming rates for the left-sided sequences [12]. It is well known that focusing attention to one of two sound streams enhances the N_1_ evoked by each tone of that stream, but typically the stimuli in these experiments were chosen such that the two streams could be readily segregated [15,16,17,18].

The influence of intentional listening on streaming has been known since at least the mid 70s: van Noorden [4] showed that the streaming threshold depends on listeners' “attentional set” within a range of ΔF and repetition rates. At the lower (upper) ΔF border lies the “fission” (temporal coherence) boundary, below (above) which listeners typically cannot hear two (one) streams. In the ambiguous range between fission and the temporal coherence boundaries, streaming is bistable and may switch spontaneously between one- and two- stream percepts [9,19]. How and if intentional listening for bistable streaming is related to selective attention is currently unclear [20,21].

The present study evaluated the hypothesis that attentional modulation of the MEG response evoked by typical streaming stimuli in the auditory cortex [9,10] is specifically observed in the ambiguous streaming range. We used a dichotic paradigm in which an oddball paradigm was presented in the right ear, and a classical streaming stimulus was presented in the left ear. Four ΔF values ranging from 0.1 to 10 semitones were chosen for the streaming stimuli based on preliminary studies with experienced listeners: The smallest ΔF value was chosen such that it would rarely produce stream segregation, i.e. the ΔF was below the fission boundary, and the largest ΔF was chosen such that it most often produced streaming, i.e. beyond the temporal coherence boundary. The two middle ΔF values were chosen to yield ambiguous streaming percepts. Based on the hypothesis outlined above, we expected attentional modulation of streaming-related activity for the ambiguous sequences (the two middle ΔF conditions), but not for the unambiguous sequences. While the streaming sequence in the left ear was periodic, the oddball sequence presented to the right ear had a random timing, such that the evoked response for the left and right ear could be independently averaged to the tone onsets in the left or right ear, respectively. This allowed us to separately evaluate the influence of whether the focus of attention was directed to the deviant-detection task in the right ear, or to the streaming task in the left ear.

## Materials and Methods

### Subjects

20 naïve listeners participated in the experiment after providing written informed consent. The mean age of the listeners was 24 years (range 20–30 years), 12 listeners were male, no listener reported a history of central or peripheral hearing disorder. Listeners were paid for their participation in the study. The study protocol was approved by the ethics committee of Heidelberg University’s medical school. One listener was excluded from the final data analysis because of extremely low performance in the right-ear distraction task and because no reliable evoked responses were obtained in response to the right-ear stimulus.

### Stimuli and procedures

**Fig 1:**
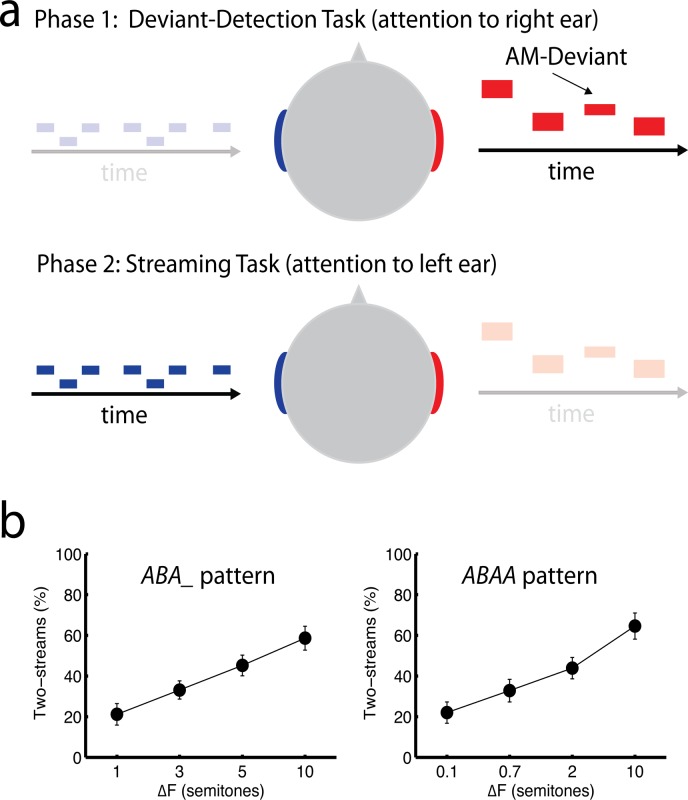
Experimental setup and behavioral results. (a) In phase one of the experiments, listeners were instructed to listen to their right ear and detect rare deviants with a 18 dB less amplitude modulation. The carrier frequency of the tones was randomized and not relevant for the task. In phase two, listeners were instructed to listen to their left ear and indicate if the alternating tone sequence presented there was perceived as one or two streams. (b) Behavioral results of the streaming task for the *ABA_* (left) and *ABAA* pattern (right) obtained in phase 2. The data plots the average percentage of two-stream ratings averaged over trials, time and subjects (N = 19; mean ± standard error).

A schematic of the experimental setup used in the study is shown in Fig. 1a. Listeners were presented with dichotic stimuli, which were comprised of a classical streaming paradigm in the left ear and an oddball paradigm in the right ear. The same stimulus sequences were presented to the listeners twice. In phase one, listeners performed a deviant-detection task: they were instructed to focus on the sequence in their right ear and indicate the occurrence of deviants by pressing a mouse button as fast as possible. They were instructed that the competing stimuli in their left ear were distractors and should be ignored. In phase two, the listeners were debriefed with regard to the stimuli on the left and instructed to indicate whether they heard one or two streams in their left ear by briefly pressing one of two mouse buttons upon each perceptual reversal. Listeners were instructed to ignore the oddball sequence in their right ear in phase two. The recording of the streaming task was started once the experimenter had the impression that the listener understood the task.

Two different tone patterns were used for the left-side streaming stimuli. One was the classical *ABA_* pattern (van Noorden, 1975). The other was a continuous *ABAA* pattern, which was previously used in a number of fMRI and MEG studies [22,23,24]. The substitution of the pause in the *ABA_* pattern with another tone in the *ABAA* pattern produces overall stronger forward suppression, thereby promoting streaming at smaller feature differences between *A* and *B* [24]. For the *ABA_* stimuli, tone duration was 100 ms and the stimulus onset asynchrony (SOA) was 150 ms. For the *ABAA* pattern, tone duration and SOA were identical at 125 ms. These different parameters were mainly chosen for comparability with previous studies [9,23]. Based on the previous studies, we expected the *ABA_* pattern to elicit prominent P_1_m and N_1_m responses due to the longer SOA between tones. The *ABAA* pattern was expected to evoke smaller P_1_m and suppressed N_1_m waves, because of the shorter SOA and additional suppression produced by the fourth tone [24].

For both patterns, four ΔF values were chosen based on informal pilot studies with experienced listeners in which the smallest ΔF almost never produced streaming, the two middle ΔF values produced bistable streaming, and the largest ΔF produced robust two-stream percepts. The ΔF was accordingly chosen to be 1, 3, 5, or 10 semitones for the *ABA_* pattern, and 0.1, 0.7, 2, or 10 semitones for the *ABAA* pattern. The frequency of *B* tones was always fixed at 1000 Hz. The *A*-tone frequency was 1060, 1189, 1335, or 1782 Hz for the *ABA_* pattern, and 1007, 1044, 1123, or 1782 Hz for the *ABAA* pattern. All tones were gated on and off by 10-ms raised-cosine windows. The streaming stimuli were presented in 32-s blocks (i.e. either 53 repetition of the *ABA_* triplet or 64 repetitions of the *ABAA* quadruplet), between which there was a 12-s pause. ΔF was held constant within a block and pseudo-randomly changed between blocks. There were four repetitions of each ΔF block for the *ABAA* quadruplet and five repetitions for the *ABA_* triplet.

In their right ear, listeners were presented with an oddball sequence of 75-ms long tones that were amplitude modulated at 100 Hz. The carrier frequency and SOA were randomized between 700 and 1,700 Hz, and 0 and 900 ms (average SOA 450 ms), respectively. The AM depth was 100% for the standards and 20% for the deviants (i.e. 18 dB less than the standards). Deviance probability was 15% without further constraints. The tone sequence in the right ear continued through the 12-s gaps separating consecutive stimulus blocks in the left ear. The stimuli were presented in four sets, with each set consisting of 16 (*ABAA*) or 20 (*ABA_*) blocks, via ER3A insert-earphones connected to 1-m custom-made plastic tubes. The first (third) set comprised *ABA_* triplets and the second (fourth) set *ABAA* quadruplets in the left ear.

### Behavioral analysis

In the deviant-detection task, all button presses registered within 1000 ms subsequent to the onset of deviant tones were counted as correct detection. Each button press outside of this time window was considered a false alarm. To estimate a valid denominator for the false alarm rate, the total oddball sequence was broken down into 1-s response intervals, not considering the 1-s response intervals belonging to target tones [25].

For the evaluation of the streaming task, the time intervals in which listeners heard one or two streams in their left ear, respectively, were determined based on the reversals indicated with the two response buttons. The temporal resolution used for this analysis was equal to the duration of one *ABA_* or *ABAA* pattern (600 ms or 500 ms). It was assumed that listeners heard one stream from the start of each 32-s long sequence up to their first indication of a two-stream response. The total time that a listener indicated to hear two streams was then divided by the overall stimulus time and expressed as percentage of the time in which two streams were perceived.

### MEG acquisition

The MEG data were acquired with a 122-channel Electa-Neuromag system in a four layer magnetically-shielded room. Prior to the recordings, four head-position-indicator coils were placed on the listeners’ forehead and mastoids and their position digitized in reference to a coordinate system defined by the two pre-auricular points and the nasion. 32 points around the head surface were additionally digitized and were later used to approximate the position of the spherical head model. The position of each coil in the MEG dewar was then measured before the recording of each of the four sets. The data were recorded at 1000 Hz sampling rate with a 330 Hz lowpass filter; no online highpass filter was applied.

### MEG analysis

MEG data were analyzed using BESA 5.1 (BESA GmbH, Munich). The evoked responses were averaged offline to (1) the onset of the triplet and quadruplet patterns presented to the left ear, separately for each ΔF and (2) the onset of the standards and deviants presented to the right ear. For left-ear stimuli, the time interval was-200 to 800 ms with respect to pattern onset, and the baseline was set in the time interval 100 ms before pattern onset. For the right-ear stimulation, the time interval was-200 to 800 ms and the baseline was set in the interval 50 ms before tone onset. Epochs containing large artifacts were rejected from further analysis using a gradient criterion, resulting in 5–10% of epochs being excluded from averaging. To establish a spatial filter for further analysis, two dipoles, one in each auditory cortex, were fit to specific peaks of the grand-average evoked response as described below. For each of the two streaming patterns, one set of dipoles was fit to the P_1_m evoked by all *B* tones, using a grand average across ΔF conditions in phase two of the experiment. For the deviant-detection experiment, the dipoles were fit to the N_1_m evoked by standards in phase one. For dipole fitting, the data were highpass filtered at 3 Hz (6 dB/oct., zero-phase-shift Butterworth filter), and lowpass filtered at 30 Hz (12dB/oct., zero-phase-shift Butterworth filter); a starting solution with dipoles in the approximate location of the auditory cortex was used and the dipoles were fit in an interval of 30 ms around the peak (of the P_1_m and N_1_m for streaming and oddball stimuli, respectively) in the global field power diagram. Approximate Talairach coordinates of dipole positions were estimated with BESA 5.1 based on the digitized head surface points.

The four resulting dipole models (one for each ear and tone pattern) were then used as a spatial filter to construct source waveforms of all single conditions, i.e. all ΔF conditions on the left and standards and deviants on the right, separately for phase one and phase two of the experiment. To model low-frequency artifacts, a principal-component analysis (PCA) was calculated in the time interval 500–600 ms (*ABA_*) and 400–500 ms (*ABAA*), i.e. in the repetition of the baseline interval. When the first PCA component was associated with a low-frequency drift rather than the repetitive peaks of the evoked response, the first PCA component was included in the spatial filter to model the artifact and avoid contamination of auditory-cortex sources. This process was performed separately for each condition. For the deviant-detection task, the PCA was calculated in the time interval 700–800 ms; the exact repetition of the baseline interval could not be chosen in this case because the inter-stimulus interval was jittered. The unfiltered source waveforms were then written to ASCII files and read into Matlab (The MathWorks, Natick, MA, USA) for further processing. Here the data were lowpass filtered at 30 Hz (12dB/oct., zero-phase-shift Butterworth filter) and the amplitudes of the P_1_m and N_1_m were measured as maxima and minima in the time interval 30–120 ms and 70–200 ms, respectively.

For the evaluation of the response elicited by the streaming stimulus, a peak-to-peak amplitude measure was used in order to mitigate the possible impact of the lacking baseline period inherent to the streaming paradigm. A similar approach was used in an earlier study to which we sought to compare the results of the present study [9]. The peak magnitudes were submitted to a repeated measures analysis of variance (ANOVA) with the factors component (P_1_m and N_1_m), hemisphere (left and right), ΔF (four levels), and experimental phase (one and two). By submitting the P_1_m and N_1_m amplitude with similar signs (i.e. multiplying the N_1_m amplitude with-1), the main effect of the analysis is equivalent to an ANOVA calculated for the peak-to-peak amplitude, but additionally allows us to evaluate if one of these components dominates the observed effects by searching for interactions including response component. The Greenhouse-Geisser correction for sphericity violations was applied to the *p* values whenever the degree of freedom in the numerator was larger than one. For the oddball sequence, where a sufficiently long baseline interval was available, the amplitudes of the P_1_m and N_1_m were separately evaluated. The peak magnitudes were submitted to a repeated measures analysis of variance (ANOVA) with the factors hemisphere (left and right) and experimental phase (one and two).

## Results

Behavioral streaming results from phase two of the experiment are shown in Fig. 1b. Across subjects, the likelihood of reporting two-stream percepts increased with ΔF (quadruplets: F_3,54_ = 11.41; p<0.0001; triplets: F_3,54_ = 19.55; p<0.0001). There was no significant difference between likelihood of two-stream responses between the two patterns. The average percentage of two-stream responses for the four conditions was 21%, 33%, 45%, and 59%. The average percentage of streaming responses for the four *ABAA* conditions was 22%, 33%, 44%, and 65%. These data concur with our pilot data, insofar as the four ΔF conditions produced similar amounts of streaming responses for the two tone patterns. However, based on pilot studies with experienced listeners, we had expected a lower percentage of streaming for the smallest (0.1 and 1 semitone) conditions (e.g., <10%), and a higher percentage of streaming for the largest (10 semitone) conditions (e.g., >80%). The deviation from the expected result is probably due to a variable and inconsistent response pattern observed in about half of the listeners, which is likely related to the comparatively short time available for streaming task instruction between the two phases of the MEG recordings. Note, however, that there is little doubt that listeners attended the left-ear streaming paradigm during phase two, which is the critical factor we sought to evaluate in the MEG data. The average number (± standard deviation) of reversals per 32-s long sequence from smallest to largest ΔF was 1.3±1.8, 1.7±1.5, 2.0±1.7, and 1.7±1.1 for the *ABA_* pattern, and 1.1±2.1, 1.4±1.0, 1.9±1.5, and 1.7±1.6 for the *ABAA* pattern.

**Fig 2:**
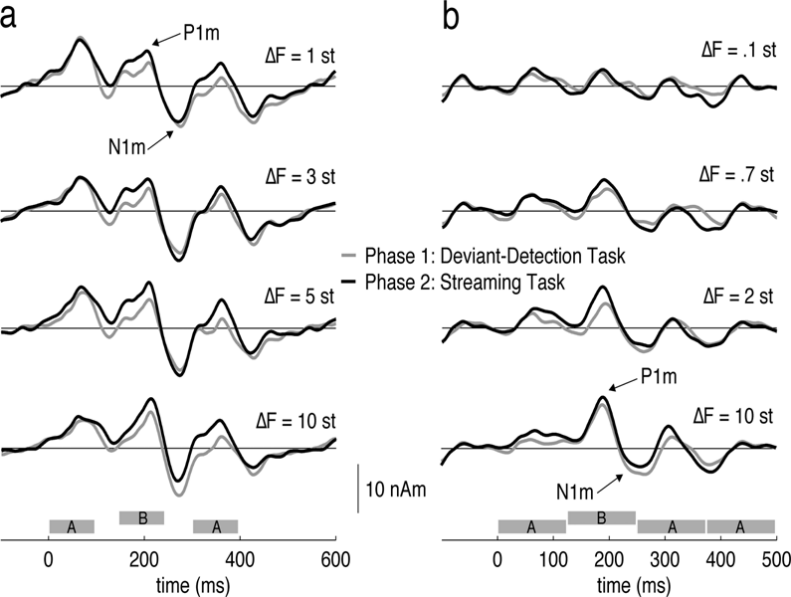
Source waveforms evoked by the left-ear streaming stimuli. (a) *ABA_* pattern (b) *ABAA* pattern. The waveforms are an average across subjects (N = 19) and hemispheres. Responses evoked in phase one are plotted in gray, those evoked in phase two of the experiment are plotted in black.

**Fig 3:**
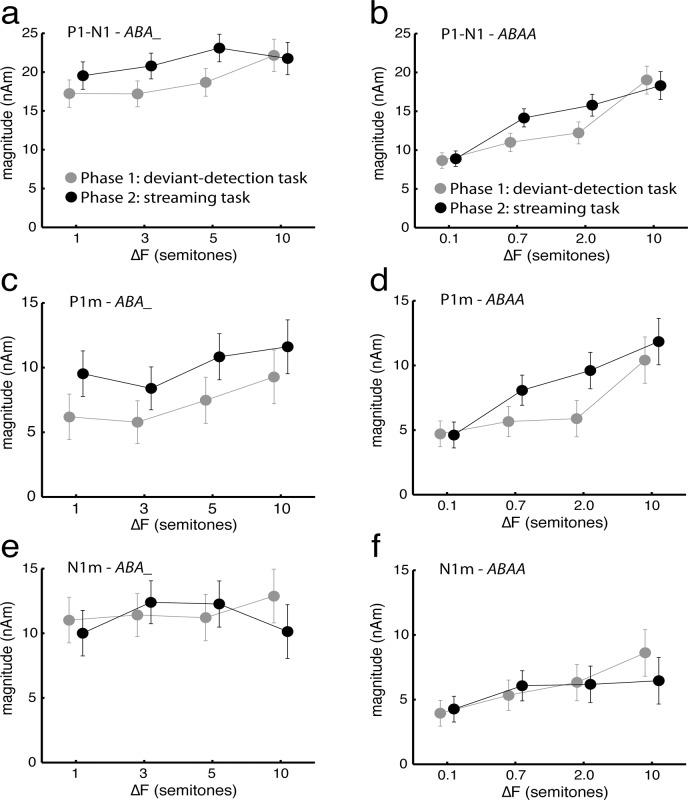
Amplitudes of the P_1_m and N_1_m evoked by the streaming stimuli. (a, b) P1m-N1m Peak-to-peak magnitude, (c, d) P1m peak magnitude, and (e, f) N1m peak magnitude. The responses were measured for the *B* tones of the *ABA_* (a, c, e) and *ABAA* (b, d, f) patterns, the frequency of which was always 1000 Hz. All values are mean source magnitudes across subjects (N = 19); error bars indicate standard errors of the mean.

Dipoles fitted to the average P_1_m evoked by the streaming stimuli were generally within the area of auditory cortex. The average Talairach coordinates (± standard deviations) in the order X, Y and Z were (*ABA_* pattern): -46±7, -19±7, 4±9 and 47±5, -18±8, 7±10. For the *ABAA* pattern, the coordinates were overall similar: -45±9, -23±7, 4±10 and 48±7, -17±5, 6±8. Fig. 2 shows the averaged evoked responses time locked to the streaming stimuli. To test our hypothesis that attention would enhance the response to the streaming stimuli for ambiguous ΔF conditions, we measured the P_1_m-N_1_m peak-to-peak amplitudes of the response evoked by the *B* tones, which was held constant at 1000 Hz such that changes in the response cannot be attributed to absolute frequency changes across conditions [9]. Fig. 3 plots the peak-to-peak amplitudes along with the separate peak amplitudes of the P_1_m and N_1_m. The statistical analysis is summarized in Table 1.

**Table 1:** Summary of ANOVA and planned comparison for the P_1_m-N_1_m amplitude evoked by the left-ear streaming sequence.

Cond	Test	DoF	F	p	Cond	Test	DoF	F	p
*ABA_*	Attn	1,18	7.29	0.0146	*ABAA*	Attn	1,18	6.52	0.020
	ΔF	3,54	7.78	0.0010		ΔF	3,54	48.34	<0.0001
	Hem	1,18	1.22	0.2844		Hem	1,18	0.14	0.7086
	Com	1,18	1.40	0.2516		Com	1,18	0.96	0.3402
	Attn*ΔF	3,54	8.70	0.0001		Attn*ΔF	3,54	4.80	0.0068
	Attn*Com	1,18	3.86	0.0650		Attn*Com	1,18	2.40	0.1389
	ΔF*Com	3,54	5.01	0.0074		ΔF*Com	3,54	0.64	0.5036
	ΔF*Hem	3,54	1.12	0.3308		ΔF*Hem	3,54	4.64	0.0123
	Attn*Hem	1,18	8.58	0.0090		Attn*Hem	1,18	3.95	0.0623
	Com*Hem	1,18	0.06	0.8124		Com*Hem	1,18	1.51	0.2345
	Attn*ΔF*Com	3,54	1.47	0.2405		Attn*ΔF*Com	3,54	1.40	0.2588
	Attn*Com*Hem	1,18	3.71	0.0700		Attn*Com*Hem	1,18	2.28	0.1483
	Attn (F1 = 1st)	1,18	4.83	0.0413		Attn (F1 = 0.1st)	1,18	0.14	0.7143
	Attn (F2 = 3st)	1,18	7.94	0.0114		Attn (F2 = 0.7st)	1,18	12.27	0.0025
	Attn (F3 = 5st)	1,18	14.27	0.0014		Attn (F3 = 2st)	1,18	7.54	0.0133
	Attn (F4 = 10st)	1,18	0.20	0.6616		Attn (F4 = 10st)	1,18	0.36	0.5579
	Attn (F1 = 1st)	1,18	8.86	0.0081		Attn (F1 = 0.1st)	1,18	0.00	0.9712
	Attn (F2 = 3st)	1,18	9.48	0.0065		Attn (F2 = 0.7st)	1,18	12.54	0.0023
	Attn (F3 = 5st)	1,18	15.89	0.0009		Attn (F3 = 2st)	1,18	10.91	0.0040
	Attn (F4 = 10st)	1,18	1.39	0.2530		Attn (F4 = 10st)	1,18	0.06	0.8065
	Attn (F1 = 1st)	1,18	0.44	0.5165		Attn (F1 = 0.1st)	1,18	0.43	0.5214
	Attn (F2 = 3st)	1,18	3.45	0.0795		Attn (F2 = 0.7st)	1,18	3.70	0.0704
	Attn (F3 = 5st)	1,18	6.45	0.0205		Attn (F3 = 2st)	1,18	3.49	0.0779
	Attn (F4 = 10st)	1,18	3.40	0.0817		Attn (F4 = 10st)	1,18	2.24	0.1518

Attn: Attention; Com: Peak Component; Cond: condition; ΔF: frequency difference; Hem: hemisphere; st: semitones; DoF: Degrees of freedom.

Our main hypothesis was that attentional enhancement of the response amplitudes would be stronger for ambiguous sequences. This effect is reflected by a significant interaction of ΔF * attention. While, there was also a main effect of attention, this effect is not homogeneous across ΔF: As can be seen in panels a and b of Fig. 3, the peak-to-peak amplitudes were indeed enhanced for the middle two ΔF values of both patterns, whereas more similar amplitudes were observed for non-ambiguous ΔF values with the exception of the smallest ΔF value of the *ABA_* pattern. The response enhancement of the two middle ΔF conditions was generally similar in both hemispheres, but somewhat more prominent on the left (cf. Table 1, S1 Fig.), which is probably the source of the hemisphere * attention interaction observed for the *ABA_* pattern.

The results further showed a significant main effect of ΔF, which reflects the frequency-selective adaptation demonstrated earlier [9]. There was no triple ΔF * attention * component interaction, which would have indicated that the ΔF * attention interaction was driven by only one of the response components.

We also evaluated the oddball paradigm presented to the right ear and the associated deviant-detection task in phase one of the experiment. The average rate of correctly-detected amplitude-modulated deviants (± standard deviation) in phase one was 61.5±15.2% when the *ABA_* pattern was played to the left ear, and 65.7±16.2% in the subsequent trial, where the *ABAA* pattern was played to the left ear. The average false alarm rate was 4.2% and 2.5%, respectively. The moderately high detection rate indicates that the task was sufficiently difficult to distract attentional resources from the streaming stimuli presented to the left ear in phase one, while the low false alarm rate confirms that the task was performed properly.

**Fig 4:**
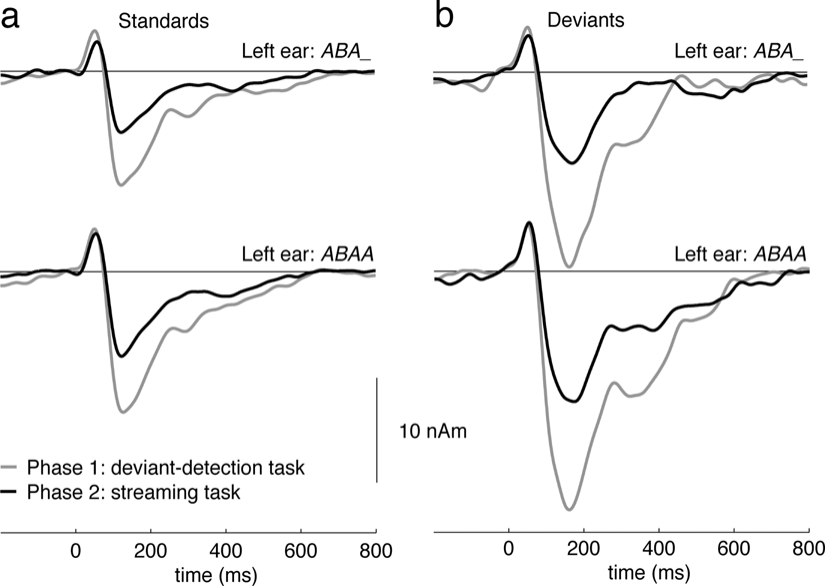
Attention effect on right-ear target detection task. Grand average source waveforms averaged across subjects (N = 19) and hemispheres. The responses are shown separately for the different left ear patterns (upper = *ABA_*; lower = *ABAA*). Responses evoked in the attended phase one are plotted in gray, responses evoked in the unattended phase two are plotted in black. The attended responses are significantly larger for standards (a) as well as deviants (b).

Dipoles fitted to the N_1_m evoked by attended standards of the oddball paradigm were within the auditory cortex. The average Talairach coordinates (± standard deviations) in the order X, Y and Z were (*ABA_* pattern in the contralateral ear): -49±7, -27±7, 6±8 and 49±5, -23±5, 7±8. When the *ABAA* pattern was presented in the contralateral ear, the coordinates were very similar: -49±6, -28±9, 6±8 and 49±6, -21±6, 5±7. Fig. 4 shows the source waveforms evoked by the oddball sequence in phases one (gray) and two (black) of the experiment. The N_1_m amplitudes were significantly larger when listeners performed the deviance-detection task in their right ear than in phase two when listeners attended the streaming stimuli on the left (F_1,18_ = 38.15; p<0.0001). As expected, deviants evoked larger N_1_m responses than standards (F_1,18_ = 57.41; p<0.0001). There was an interaction of deviance * attention (F_1,18_ = 18.04; p = 0.0005), due to the stronger response enhancement for attended compared to non-attended deviants. These findings further confirm that listeners attended the right ear in phase one and the left ear in phase two.

Further statistical results show that N_1_m evoked by the oddball sequence were overall stronger in the trial where the *ABAA* pattern was played to the left ear (F_1,18_ = 6.96; p = 0.0167). This effect cannot be explained by response habituation, because the *ABAA* pattern was always presented subsequently to the *ABA_* pattern. There was no significant main effect or interaction including hemisphere in the analysis of N_1_m amplitudes. The average N_1_m latency was 144.7 ms for phase 1 and 144.4 ms in phase 2. No significant difference between phases was observed for the P_1_m amplitude (F_1,18_ = 2.36; p = 0.1419), however, there was a significant effect of deviance (F_1,18_ = 13.50; p = 0.0017) caused by stronger P_1_m for deviants compared to standards.

## Discussion

Our results show that the P_1_m-N_1_m evoked by rapidly alternating tone patterns is not significantly modulated by attention when ΔF is in the non-ambiguous range for streaming. Even when attention is distracted, the response is significantly stronger for large ΔF associated with two-streams percepts than for small ΔF associated with one-stream percepts. The evoked response is more variable in an intermediate ΔF range, where the perception of the sequence is typically ambiguous. Only in this range did we observe a response enhancement of the P_1_m-N_1_m response when attention was directed towards the streaming task. These results are consistent with the hypothesis that attention modulates streaming-related activity in auditory cortex more strongly for ambiguous streaming conditions. While the order was not randomized in this experiment to keep listeners naïve with respect to the streaming task, it is unlikely that the task order had a relevant effect on the results: Response habituation has been shown to reduce the N_1_m in the course of tens of minutes. While N_1_m reduction may conversely lead to some enhancement of the P_1_m—probably due to reduced cancelation—the P_1_m-N_1_m peak-to-peak-amplitude typically decreases over time [26]. Since the streaming paradigm was attended in phase 2, response reduction of the P_1_m-N_1_m could have been caused by habituation, whereas it is unlikely that the observed response enhancement could be due to the fixed task order. Moreover, order effects would not be expected to selectively modify the two middle ΔF conditions. These results are in line with a model of streaming which is largely automatic provided that distinct sources are sufficiently separated in acoustic feature space, but which can nevertheless be modulated by attention when presented with ambiguous acoustic input. This conclusion is based on the assumption that the evoked response is larger when the two streams show less interaction, because of selective adaptation, thereby supporting their grouping in separate streams [9,10,11,23,27,28]. Attention may bias the specificity of selective adaptation, decrease the interaction between streams, and enhance their probability to be perceived as two segregated streams. Interestingly, the effect was not stronger in the auditory cortex contralateral to the stimulation but in the ipsilateral, left auditory cortex. This finding could potentially be related to previous reports of left-hemisphere dominance for attentional, schema-based stream segregation [29].

Snyder et al. [10] did not find a ΔF-selective modulation when they compared EEG responses evoked by a streaming stimulus while listeners either rated their streaming perception or attended to a visual distraction task. However, close inspection of Figure 5 from that study shows a trend for selective modulation of their ambiguous ΔF = 4 semitones condition. While it has been shown that visual distraction can also reduce streaming build-up [30], the effect is weaker, and it could be that this is the reason why the effect is observed with an auditory distractor in the present study but not with the visual distractor used by Snyder *et al*. In their streaming experiment with neglect patients, Carlyon et al. [12] showed that streaming probability was lower in the neglected left ear than in the right ear. Interestingly, this effect was only observed for intermediate, ambiguous frequency conditions, similar to the attentional modulation observed in the present study. If attention was generally required for streaming [12], one would not expect such a difference between higher and intermediate ΔF regions.

At this point, our result cannot prove the model of early modulation of frequency specificity in the ambiguous streaming range. For example, it could be that the ambiguous sequences draw more attention towards the streaming stimuli because the classification is harder than for the less ambiguous conditions. Alternatively, it could be that the more frequent occurrence of perceptual reversals in the ambiguous conditions leads to less adaptation of the evoked response. It is unlikely that the reversals themselves, or associated motor activity, are related to the response enhancement, since the MEG response is time locked to the single tones, and not to the reversals as shown for activity in fMRI [31,32]. Moreover, the number of reversals was overall low in the present experiment.

An important question is how the attentional modulation of activity evoked by the streaming paradigm is related to response enhancement in auditory cortex observed in the context of selective attention [15,17,18]. This effect is replicated here by the oddball paradigm presented to the right ear. Attentional enhancement during oddball paradigms mainly involves an enlarged negativity in the N_1_m latency range, referred to as the negative difference wave (Nd) [16,17], comprised of mostly low-frequency activity (i.e. below 6 Hz). In comparison to the task-dependent modulation in the context of the streaming paradigm, the N_1_m enhancement during the oddball paradigm is much more prominent. A similar enhancement irrespective of ΔF might reasonably be expected for the attended streaming sequence. We did not observe such a large N_1_m enhancement for the streaming stimuli, likely due to the periodic presentation of tones at 6.7 Hz (*ABA_*) and 8 Hz (*ABAA*), which evokes a narrow-band steady-state response with maxima at the fundamental presentation rate and its harmonics. If the Nd response was evoked by each of the tones, its low-frequency nature would result in a linear negative drift but would not be reflected by the time-locked evoked response. Such an attention-dependent negative drift for streaming stimuli has indeed been shown by others and was independent of the ΔF used [10]. The negative trend would have been removed by the analysis applied to our data, however, and is generally difficult to record in an environment with low-frequency noise, as is present at our MEG site.

The situation might be expected to be different when only the *B*-tone stream is considered, given that the *B*-tone repetition rate was 2 Hz (*ABAA*) or 1.7 Hz (*ABA_*). Such low repetition rates might leave the Nd partly intact as a time-locked response. If listeners attended selectively to the *B*-tone stream, one would predict an Nd evoked by *B* tones but not *A* tones. Such a response could probably not be expected for a stimulus below the fission boundary, because there is no segregated *B*-tone stream in this case to which attention could be selectively directed. For the ΔF of 10 semitones, however, selective attention would be expected to produce enhancement to the response evoked by *B* tones [33]. The latter prediction does not match the findings of the present study, likely because listeners were not instructed to listen selectively to the *B*-tone stream, but to listen to the left-ear streaming pattern in total.

Modulation of the P_1_m-N_1_m has been previously observed when one- and two-stream percepts were compared in bistable sequences [9]. While listeners attended continuously to the streaming paradigm in the former study, the contrast in the present study is between the attended and unattended pattern. It is nevertheless conceivable that the attentional modulation time locked to the *B* tones in the sequence could be related to a similar mechanism. In the context of bistable perception, it has been suggested that either the P_1_m (and N_1_m) are directly modulated [9]—or that there is an additional, more specific response component [34,35]. In any case, there is converging evidence that activity in the latency range of 60–140 ms is more positive when two streams are perceived [9,10,21,34,35].

In summary, the present study provides support for the hypothesis that focused attention selectively modulates the activity evoked by streaming stimuli when the percept is ambiguous. The same modulation was not consistently observed for conditions where the ΔF was either (i) too small for streaming to occur or (ii) too large for streaming not to occur. These data therefore raise the possibility that, while attention can support stream segregation, it may only play a role for perceptually bistable stimulus configurations. The constraint for accessing this question behaviorally is the problem of measuring streaming in the absence of selective attention to the test sound [12]. Several other aspects remain to be explored in more detail, such as the relationship between the effect observed here and response enhancement in the auditory cortex for selectively attended streams [15,17,18], as well as the interaction of the auditory cortex with other brain areas such as the intra-parietal sulcus [36,37].

## Supporting Information

S1 Fig Amplitudes of the P_1_m-N_1_m evoked by the streaming stimuli. The data in this figure are similar to panels a and b of Fig. 3 in the main manuscript, but the peak-to-peak amplitude is plotted separately for the left and right auditory cortex.
(TIF)
