# Supplementary figures and images for: Interaction of Streaming and Attention in Human Auditory Cortex
Source: PLoS One. 2015 Mar 18;10(3):e0118962. doi: 10.1371/journal.pone.0118962 (PMC4364770; doi:10.1371/journal.pone.0118962)

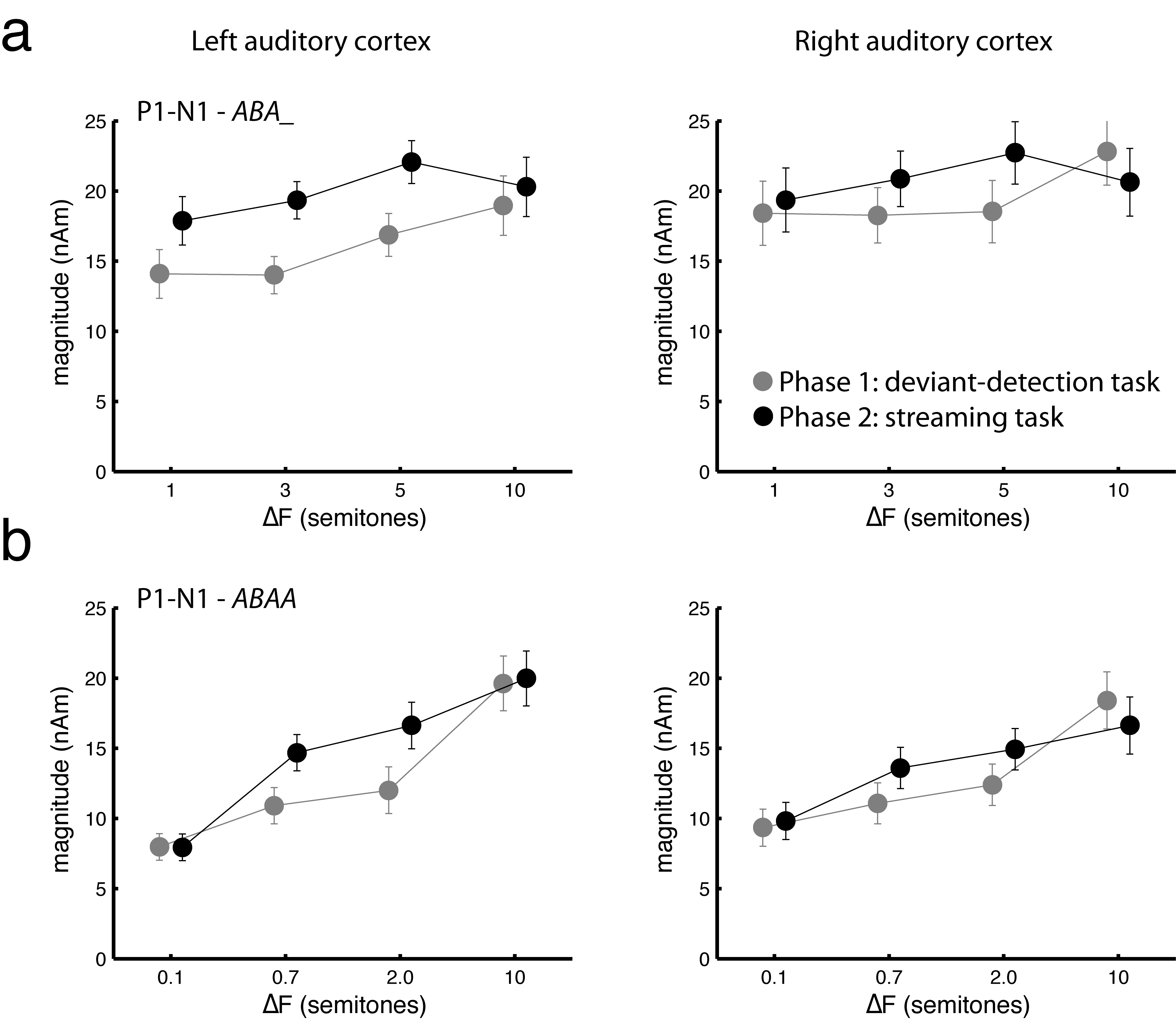

Supplement: S1 Fig — The data in this figure are similar to panels a and b of Fig. 3 in the main manuscript, but the peak-to-peak amplitude is plotted separately for the left and right auditory cortex. (TIF) [file pone.0118962.s001.tif]
